# Reproduction, genetics, and health – a topic with implications far beyond infertility

**DOI:** 10.1515/medgen-2024-2037

**Published:** 2024-09-06

**Authors:** Margot J. Wyrwoll, Frank Tüttelmann

**Affiliations:** University of Edinburgh Centre for Regenerative Medicine 5 Little France Drive EH16 4UU Edinburgh United Kingdom; University and University Hospital Münster Centre of Medical Genetics Vesaliusweg 12–14 48149 Münster Germany

Reproductive genetics is an emerging field that has long been considered less relevant than, for example, cancer genetics or neurogenetics. This may be because infertility is not a life-threatening condition, although it can be caused by ovarian or testicular cancer. Nevertheless, infertility is a disease and is defined by the World Health Organization (WHO) as the failure to achieve a pregnancy after at least twelve months of regular unprotected intercourse [1]. What distinguishes infertility from other diseases is that it regularly affects couples rather than individuals. Indeed, affecting around 17 % of all couples worldwide, it is one of the most prevalent diseases in our society. However, for most infertile patients, medical care is not (fully) covered by the health insurance, leaving many infertile patients to bear a significant financial burden. This creates a situation of social injustice, as not all couples are able to afford the costly treatments required.

As a consequence of recent scientific advances, we are beginning to understand the genetic origins of infertility, allowing for more precise and individualised treatments. Moreover, an increasing number of associations between infertility and other diseases are being revealed, demonstrating the importance of further research to be conducted in order to elucidate those links in more detail. This issue addresses the causes of male and female infertility, the approach that should be taken with affected couples during genetic counselling, and the current state of knowledge regarding concomitant diseases.

The Special Issue commences with a discussion of the *Genetics of female and male infertility*. This presents established knowledge and recent developments concerning genetic causes of infertility. While chromosomal aberrations have been known for a long time as cause of infertility in both sexes, monogenic causes have only been identified in the last years and continue to be discovered, continuously increasing the diagnostic yield. The identification of these causes has consequences for the treatment in many cases and patients can be counselled precisely before undergoing treatment.

The second article, *Psychological aspects of infertility*, sheds light on the disparate impact of infertility and medically assisted reproduction (MAR) have on females and males. It also presents a summary of the coping strategies employed by affected couples and their long-term outcomes, as well as recommendations for improving the efficacy of counselling for these couples. It is of paramount importance to recognise that these couples frequently experience stress levels comparable to those experienced by individuals facing life-threatening diseases or the loss of a family member [2].

The third article, *Genetics and Reproductive Health*, provides an overview on the links between infertility and other diseases. In particular, the increased risks of cancer and cardiovascular diseases must be considered, although the underlying mechanisms of theses associations are poorly understood to date. Furthermore, this article points out the health risks for children conceived by MAR. While the increased risks of birth defects have been known for many years, only recently have we begun to observe the risks in the later lives of these persons.

This Special Issue would not have been possible without the invaluable contributions of the authors, whose work we believe will facilitate a deeper understanding of reproductive genetics.
